# Supplementary material for: Shifting respiratory pathogens: Post-COVID-19 trends in community-acquired infections in underserved communities
Source: PLoS One. 2025 Aug 22;20(8):e0329481. doi: 10.1371/journal.pone.0329481 (PMC12373226; doi:10.1371/journal.pone.0329481)
Supplement: S1 Table — (DOCX) [file pone.0329481.s001.docx]

Table S1. Sociodemographic and risk factor determinants of acute community-acquired upper respiratory infections in the study population.

|  | **Univariate analysis** | | **Model 1^i^** | | | **Model 2^ii^** | | |
| --- | --- | --- | --- | --- | --- | --- | --- | --- |
|  | **Infection** | |  | | |  | | |
|  | % | ***P*** | **adj. OR** | **95%CI** | ***P*** | **adj. OR** | **95%CI** | ***P*** |
| **Age class** |  |  |  |  |  |  |  |  |
| ≤5 years | **89.6** | **<0.001** |  |  |  |  |  |  |
| 6-17 years | **62.2** |  | 0.35 | 0.09-1.26 | 0.110 |  |  |  |
| ≥18 years | **59.0** |  | 0.58 | 0.12-2.77 | 0.497 |  |  |  |
| **Sex** |  |  |  |  |  |  |  |  |
| Female | 68.9 | 1.00 |  |  |  |  |  |  |
| Male^1^ | 69.5 |  |  |  |  |  |  |  |
| **Region** |  |  |  |  |  |  |  |  |
| Urban | 69.2 | 0.396 |  |  |  |  |  |  |
| Rural | 76.6 |  |  |  |  |  |  |  |
| **Educational level** |  |  |  |  |  |  |  |  |
| High school or above | **59.8** | **<0.001** | **0.26** | **0.08-0.76** | **0.016** | **0.25** | **0.11-0.53** | **<0.001** |
| Less than High School^1^ | **81.4** |  |  |  |  |  |  |  |
| **Marital status** |  |  |  |  |  |  |  |  |
| Single | **76.4** | **0.003** | 0.78 | 0.35-1.73 | 0.546 |  |  |  |
| Married^1^ | **58.8** |  |  |  |  |  |  |  |
| **Environment** |  |  |  |  |  |  |  |  |
| Overcrowded | 73.4 | 0.352 |  |  |  |  |  |  |
| Regular^1^ | 67.4 |  |  |  |  |  |  |  |
| **Vaccinated against influenza** |  |  |  |  |  |  |  |  |
| Yes | 63.6 | 0.734 |  |  |  |  |  |  |
| No^1^ | 69.5 |  |  |  |  |  |  |  |
| **Citizenship** |  |  |  |  |  |  |  |  |
| Lebanese | 68.9 | 0.242 |  |  |  |  |  |  |
| Syrian or Palestinian refugee^1^ | 86.7 |  |  |  |  |  |  |  |
| **Season** |  |  |  |  |  |  |  |  |
| Fall^1^ | **73.3** | **0.002** |  |  |  |  |  |  |
| Other seasons | **53.0** |  | 0.71 | 0.29-1.74 | 0.447 | 0.48 | 0.20-1.17 | 0.107 |
| **Cough** |  |  |  |  |  |  |  |  |
| Yes | **73.6** | **0.017** | 1.65 | 0.77-3.53 | 0.197 |  |  |  |
| No^1^ | **58.5** |  |  |  |  |  |  |  |
| **Fever** |  |  |  |  |  |  |  |  |
| Yes | **74.5** | 0.073 | 1.93 | 0.95-3.96 | 0.070 | 1.86 | 0.93-3.77 | 0.081 |
| No^1^ | **64.5** |  |  |  |  |  |  |  |
| **Nausea** |  |  |  |  |  |  |  |  |
| Yes | 72.7 | 0.754 |  |  |  |  |  |  |
| No^1^ | 69.1 |  |  |  |  |  |  |  |
| **Vomiting** |  |  |  |  |  |  |  |  |
| Yes | 76.5 | 0.467 |  |  |  |  |  |  |
| No^1^ | 68.7 |  |  |  |  |  |  |  |
| **Dyspnea** |  |  |  |  |  |  |  |  |
| Yes | 75.2 | 0.114 | 1.56 | 0.73-3.45 | 0.259 | 1.81 | 0.84-4.06 | 0.138 |
| No^1^ | 66.0 |  |  |  |  |  |  |  |
| **Headache** |  |  |  |  |  |  |  |  |
| Yes | 63.4 | 0.104 | 1.62 | 0.71-3.76 | 0.256 |  |  |  |
| No^1^ | 73.0 |  |  |  |  |  |  |  |
| **Malaise** |  |  |  |  |  |  |  |  |
| Yes | 68.5 | 0.828 |  |  |  |  |  |  |
| No^1^ | 70.3 |  |  |  |  |  |  |  |
| **Moderate-to-severe diarrhea** |  |  |  |  |  |  |  |  |
| Yes | 59.4 | 0.180 | 0.43 | 0.15-1.21 | 0.107 | 0.43 | 0.15-1.25 | 0.117 |
| No^1^ | 72.5 |  |  |  |  |  |  |  |
| **Itchy nose or throat** |  |  |  |  |  |  |  |  |
| Yes | 74.6 | 0.465 |  |  |  |  |  |  |
| No^1^ | 68.7 |  |  |  |  |  |  |  |
| **Muscle pain** |  |  |  |  |  |  |  |  |
| Yes | **57.7** | **0.001** | 0.69 | 0.30-1.58 | 0.381 |  |  |  |
| No^1^ | **76.3** |  |  |  |  |  |  |  |
| **Runny nose** |  |  |  |  |  |  |  |  |
| Yes | **75.6** | **0.011** | **3.72** | **1.88-7.63** | **<0.001** | **3.98** | **1.99-8.30** | **0.001** |
| No^1^ | **61.4** |  |  |  |  |  |  |  |
| **Wheezing** |  |  |  |  |  |  |  |  |
| Yes | **91.2** | **0.007** | **7.71** | **1.76-57.8** | **0.017** | **7.57** | **1.80-54.0** | **0.015** |
| No^1^ | **66.8** |  |  |  |  |  |  |  |
| **Chest pain** |  |  |  |  |  |  |  |  |
| Yes | **58.3** | **0.028** | **0.30** | **0.11-0.75** | **0.012** | **0.35** | **0.14-0.81** | **0.015** |
| No^1^ | **72.9** |  |  |  |  |  |  |  |
| **Asthma** |  |  |  |  |  |  |  |  |
| Yes | 54.2 | 0.115 | 0.41 | 0.10-1.65 | 0.202 |  |  |  |
| No^1^ | 71.7 |  |  |  |  |  |  |  |
| **Hypertension** |  |  |  |  |  |  |  |  |
| Yes | 54.2 | 0.115 | 1.27 | 0.31-5.83 | 0.743 |  |  |  |
| No^1^ | 71.7 |  |  |  |  |  |  |  |
| **Sinusitis** |  |  |  |  |  |  |  |  |
| Yes | **37.9** | **<0.001** | **0.31** | **0.11-0.87** | **0.027** | **0.21** | **0.07-0.59** | **0.004** |
| No^1^ | **73.7** |  |  |  |  |  |  |  |
| **Sore throat** |  |  |  |  |  |  |  |  |
| Yes | 70.0 | 0.927 |  |  |  |  |  |  |
| No^1^ | 68.9 |  |  |  |  |  |  |  |

*Determinants of infection were predicted using univariate and multivariable analysis. ^i^The variables tested by univariate analysis that had a P-value ≤ 0.20 were included in Model 1 (multivariable logistic regression analysis). ^ii^In Model 2, a backward logistic regression model was created including only complete cases. ^1^Reference group. Bold and red values indicate statistically significant results.
