# Supplementary material for: Shifting respiratory pathogens: Post-COVID-19 trends in community-acquired infections in underserved communities
Source: PLoS One. 2025 Aug 22;20(8):e0329481. doi: 10.1371/journal.pone.0329481 (PMC12373226; doi:10.1371/journal.pone.0329481)
Supplement: S2 Table — (DOCX) [file pone.0329481.s002.docx]

Table S2. Determinants of pathogens among patients suffering from acute community-acquired upper respiratory infections using multivariable logistic regression models in Lebanon.

|  | **Human rhinovirus/enterovirus** | | | | **Influenza A Virus** | | | | **Respiratory syncytial virus** | | | | **SARS-CoV-2** | | | | **Parainfluenza Virus** | | | |
| --- | --- | --- | --- | --- | --- | --- | --- | --- | --- | --- | --- | --- | --- | --- | --- | --- | --- | --- | --- | --- |
|  | **Model 1**^i^ | | **Model 2**^ii^ | | **Model 1**^i^ | | **Model 2**^ii^ | | **Model 1**^i^ | | **Model 2**^ii^ | | **Model 1**^i^ | | **Model 2**^ii^ | | **Model 1**^i^ | | **Model 2**^ii^ | |
|  | **adj. OR (IC95%)** | **P-value** | **adj. OR (IC95%)** | **P-value** | **adj. OR (IC95%)** | **P-value** | **adj. OR (IC95%)** | **P-value** | **adj. OR (IC95%)** | **P-value** | **adj. OR (IC95%)** | **P-value** | **adj. OR (IC95%)** | **P-value** | **adj. OR (IC95%)** | **P-value** | **adj. OR (IC95%)** | **P-value** | **adj. OR (IC95%)** | **P-value** |
| **Age** |  |  |  |  |  |  |  |  |  |  |  |  |  |  |  |  |  |  |  |  |
| ≤5 years^1^ |  |  |  |  |  |  |  |  |  |  |  |  |  |  |  |  |  |  |  |  |
| 6-17 years | 0.57 (0.17-1.73) | 0.335 |  |  | **4.61 (1.34-17.3)** | **0.018** | 3.52 (0.99-13.2) | 0.054 | 0.35 (0.07-1.33) | 0.154 |  |  | 0.29 (0.01-2.83) | 0.352 |  |  | 0.58 (0.08-2.73) | 0.523 |  |  |
| ≥18 years | 0.37 (0.11-1.20) | 0.105 |  |  | 2.77 (0.61-12.8) | 0.187 | 2.95 (0.77-1.21) | 0.122 | 0.52 (0.05-4.36) | 0.562 |  |  | 0.70 (0.06-6.04) | 0.765 |  |  | 0.55 (0.12-2.21) | 0.418 |  |  |
| **Region** |  |  |  |  |  |  |  |  |  |  |  |  |  |  |  |  |  |  |  |  |
| Rural | 1.11 (0.49-2.43) | 0.802 |  |  |  |  |  |  |  |  |  |  |  |  |  |  |  |  |  |  |
| Urban^1^ |  |  |  |  |  |  |  |  |  |  |  |  |  |  |  |  |  |  |  |  |
| **Educational level** |  |  |  |  |  |  |  |  |  |  |  |  |  |  |  |  |  |  |  |  |
| High school or above | 1.25 (0.46-3.60) | 0.663 |  |  | **0.23 (0.07-0.78)** | **0.021** | **0.23 (0.06-0.76)** | **0.019** | 0.45 (0.07-2.67) | 0.382 | **0.29 (0.10-0.76)** | **0.017** | 3.47 (0.54-38.3) | 0.247 | 3.55 (1.09-14.7) | 0.051 |  |  |  |  |
| Less than High School^1^ |  |  |  |  |  |  |  |  |  |  |  |  |  |  |  |  |  |  |  |  |
| **Marital status** |  |  |  |  |  |  |  |  |  |  |  |  |  |  |  |  |  |  |  |  |
| Single |  |  |  |  | 0.83 (0.26-2.58) | 0.742 |  |  | 1.03 (0.18-5.48) | 0.968 |  |  |  |  |  |  | 1.99 (0.33-15.8) | 0.463 | 4.06 (0.70-77.2) | 0.196 |
| Married^1^ |  |  |  |  |  |  |  |  |  |  |  |  |  |  |  |  |  |  |  |  |
| **Vaccinated against influenza** |  |  |  |  |  |  |  |  |  |  |  |  |  |  |  |  |  |  |  |  |
| Yes |  |  |  |  | 0.00 (0-2.4e^26^) | 0.992 | 0.00 (0-2.2e^20^) | 0.993 |  |  |  |  |  |  |  |  |  |  |  |  |
| No^1^ |  |  |  |  |  |  |  |  |  |  |  |  |  |  |  |  |  |  |  |  |
| **Citizenship** |  |  |  |  |  |  |  |  |  |  |  |  |  |  |  |  |  |  |  |  |
| Syrian or Palestinian refugee |  |  |  |  | 3.86 (0.76-21.6) | 0.107 | 4.24 (0.81-24.4) | 0.090 |  |  |  |  |  |  |  |  | 4.61 (0.74-24.1) | 0.078 | **7.22 (1.19-37.0)** | **0.020** |
| Lebanese^1^ |  |  |  |  |  |  |  |  |  |  |  |  |  |  |  |  |  |  |  |  |
| **Season** |  |  |  |  |  |  |  |  |  |  |  |  |  |  |  |  |  |  |  |  |
| Other seasons |  |  |  |  | 0.00 (0.0-4.7e^12^) | 0.987 | 0.00 (0-8.0e^17^) | 0.990 | 0.00 (0.0-2.0e^18^) | 0.989 | 0.00 (0-6.1e^28^) | 0.991 |  |  |  |  |  |  |  |  |
| Fall^1^ |  |  |  |  |  |  |  |  |  |  |  |  |  |  |  |  |  |  |  |  |
| **Cough** |  |  |  |  |  |  |  |  |  |  |  |  |  |  |  |  |  |  |  |  |
| Yes |  |  |  |  | 3.04 (1.06 -10.4) | 0.052 | **3.33 (1.18-11.2)** | **0.034** | 1.09 (0.35-3.82) | 0.885 |  |  | 0.65 (0.23-1.91) | 0.420 |  |  |  |  |  |  |
| No^1^ |  |  |  |  |  |  |  |  |  |  |  |  |  |  |  |  |  |  |  |  |
| **Fever** |  |  |  |  |  |  |  |  |  |  |  |  |  |  |  |  |  |  |  |  |
| Yes | **0.50 (0.26-0.94)** | **0.033** | 0.57 (0.31-1.05) | 0.072 | **8.07 (3.32-22.7)** | **<0.001** | **7.63 (3.19-20.9)** | **<0.001** |  |  |  |  |  |  |  |  |  |  |  |  |
| No^1^ |  |  |  |  |  |  |  |  |  |  |  |  |  |  |  |  |  |  |  |  |
| **Nausea** |  |  |  |  |  |  |  |  |  |  |  |  |  |  |  |  |  |  |  |  |
| Yes |  |  |  |  | 0.61 (0.13-2.39) | 0.499 |  |  | 0.64 (0.07-3.67) | 0.639 |  |  |  |  |  |  |  |  |  |  |
| No^1^ |  |  |  |  |  |  |  |  |  |  |  |  |  |  |  |  |  |  |  |  |
| **Vomiting** |  |  |  |  |  |  |  |  |  |  |  |  |  |  |  |  |  |  |  |  |
| Yes |  |  |  |  | **5.99 (1.37-28.1)** | **0.018** | **4.06 (1.39-12.4)** | **0.011** | 0.38 (0.02-3.31) | 0.442 |  |  |  |  |  |  |  |  |  |  |
| No^1^ |  |  |  |  |  |  |  |  |  |  |  |  |  |  |  |  |  |  |  |  |
| **Dyspnea** |  |  |  |  |  |  |  |  |  |  |  |  |  |  |  |  |  |  |  |  |
| Yes |  |  |  |  |  |  |  |  | 2.31 (0.94-5.79) | 0.069 | **2.69 (1.11-6.66)** | **0.029** |  |  |  |  |  |  |  |  |
| No^1^ |  |  |  |  |  |  |  |  |  |  |  |  |  |  |  |  |  |  |  |  |
| **Headache** |  |  |  |  |  |  |  |  |  |  |  |  |  |  |  |  |  |  |  |  |
| Yes | 1.05 (0.45-2.44) | 0.918 |  |  |  |  |  |  | 0.63 (0.07-3.65) | 0.628 |  |  |  |  |  |  |  |  |  |  |
| No^1^ |  |  |  |  |  |  |  |  |  |  |  |  |  |  |  |  |  |  |  |  |
| **Malaise** |  |  |  |  |  |  |  |  |  |  |  |  |  |  |  |  |  |  |  |  |
| Yes |  |  |  |  |  |  |  |  | 1.04 (0.29-3.47) | 0.948 |  |  |  |  |  |  |  |  |  |  |
| No^1^ |  |  |  |  |  |  |  |  |  |  |  |  |  |  |  |  |  |  |  |  |
| **Moderate-to-severe diarrhea** |  |  |  |  |  |  |  |  |  |  |  |  |  |  |  |  |  |  |  |  |
| Yes | 0.50 (0.14-1.50) | 0.251 |  |  |  |  |  |  |  |  |  |  |  |  |  |  |  |  |  |  |
| No^1^ |  |  |  |  |  |  |  |  |  |  |  |  |  |  |  |  |  |  |  |  |
| **Muscle pain** |  |  |  |  |  |  |  |  |  |  |  |  |  |  |  |  |  |  |  |  |
| Yes | 1.31 (0.57-3.03) | 0.531 |  |  |  |  |  |  | 0.16 (0.01-1.18) | 0.120 | **0.08 (0.0-0.42)** | **0.017** |  |  |  |  |  |  |  |  |
| No^1^ |  |  |  |  |  |  |  |  |  |  |  |  |  |  |  |  |  |  |  |  |
| **Runny nose** |  |  |  |  |  |  |  |  |  |  |  |  |  |  |  |  |  |  |  |  |
| Yes | **3.73 (1.97-7.38)** | **<0.001** | **4.16 (2.19-8.28)** | **<0.001** |  |  |  |  | 0.45 (0.18-1.08) | 0.079 | **0.37 (0.15-0.90)** | **0.031** |  |  |  |  | **10.7 (2.03-197)** | **0.025** | 7.05 (1.26-132) | 0.069 |
| No^1^ |  |  |  |  |  |  |  |  |  |  |  |  |  |  |  |  |  |  |  |  |
| **Wheezing** |  |  |  |  |  |  |  |  |  |  |  |  |  |  |  |  |  |  |  |  |
| Yes |  |  |  |  |  |  |  |  | 1.18 (0.34-3.74) | 0.787 |  |  | **5.20 (1.36-19.5)** | **0.013** | **6.22 (1.58-24.8)** | **0.008** |  |  |  |  |
| No^1^ |  |  |  |  |  |  |  |  |  |  |  |  |  |  |  |  |  |  |  |  |
| **Chest pain** |  |  |  |  |  |  |  |  |  |  |  |  |  |  |  |  |  |  |  |  |
| Yes | 0.55 (0.23-1.25) | 0.169 | **0.43 (0.19-0.91)** | **0.034** |  |  |  |  |  |  |  |  | **0.13 (0.02-0.58)** | **0.018** | **0.12 (0.02-0.56)** | **0.018** |  |  |  |  |
| No^1^ |  |  |  |  |  |  |  |  |  |  |  |  |  |  |  |  |  |  |  |  |
| **Asthma** |  |  |  |  |  |  |  |  |  |  |  |  |  |  |  |  |  |  |  |  |
| Yes |  |  |  |  |  |  |  |  |  |  |  |  |  |  |  |  | **4.28 (0.92-16.9)** | **0.046** | 3.94 (0.66-18.6) | 0.099 |
| No^1^ |  |  |  |  |  |  |  |  |  |  |  |  |  |  |  |  |  |  |  |  |
| **Sinusitis** |  |  |  |  |  |  |  |  |  |  |  |  |  |  |  |  |  |  |  |  |
| Yes | 0.45 (0.12-1.36) | 0.189 | 0.43 (0.11-1.28) | 0.157 | 0.33 (0.02-1.95) | 0.309 |  |  |  |  |  |  |  |  |  |  |  |  |  |  |
| No^1^ |  |  |  |  |  |  |  |  |  |  |  |  |  |  |  |  |  |  |  |  |
| **Sore throat** |  |  |  |  |  |  |  |  |  |  |  |  |  |  |  |  |  |  |  |  |
| Yes | 0.91 (0.47-1.79) | 0.793 |  |  |  |  |  |  |  |  |  |  | 2.86 (1.01-8.83) | 0.055 | 2.68 (0.93-8.49) | 0.076 |  |  |  |  |
| No^1^ |  |  |  |  |  |  |  |  |  |  |  |  |  |  |  |  |  |  |  |  |

^i^The variables tested by univariate analysis that had a P-value ≤ 0.20 were included as explanatory variables in Model 1 (multivariable logistic regression analysis). ^ii^In model 2, a backward logistic regression model was created including only complete cases. ^1^Reference group. Bold and red values indicate statistically significant results.
