# Supplementary material for: Shifting respiratory pathogens: Post-COVID-19 trends in community-acquired infections in underserved communities
Source: PLoS One. 2025 Aug 22;20(8):e0329481. doi: 10.1371/journal.pone.0329481 (PMC12373226; doi:10.1371/journal.pone.0329481)
Supplement: S3 Table — (DOCX) [file pone.0329481.s003.docx]

Table S3. Association between common viruses (Prevalence > 4%) among patients suffering from acute community-acquired upper respiratory infections using multivariable logistic regression models in Lebanon.

|  | **Model 1^i^** | | | **Model 2^ii^** | | |
| --- | --- | --- | --- | --- | --- | --- |
|  | **adj. OR** | **95% CI** | **P-value** | **adj. OR** | **95% CI** | **P-value** |
| **Human rhinovirus/enterovirus** |  |  |  |  |  |  |
| **Influenza A Virus** | **0.25** | **0.10-0.53** | **<0.001** | **0.25** | **0.10-0.54** | **0.001** |
| **Respiratory syncytial virus** | 0.71 | 0.30-1.55 | 0.411 |  |  |  |
| **SARS-CoV-2** | **0.20** | **0.03-0.73** | **0.036** | **0.21** | **0.03-0.75** | **0.039** |
| **Parainfluenza Virus** | 1.01 | 0.31-2.90 | 0.981 |  |  |  |
| **Influenza A Virus** |  |  |  |  |  |  |
| **Human rhinovirus/enterovirus** | **0.24** | **0.10-0.53** | **<0.001** | **0.24** | **0.10-0.53** | **0.001** |
| **Respiratory syncytial virus** | 0.39 | 0.11-1.06 | 0.095 | 0.40 | 0.11-1.06 | 0.096 |
| **SARS-CoV-2** | 0.13 | 0.01-0.66 | 0.051 | 0.13 | 0.01-0.67 | 0.053 |
| **Parainfluenza Virus** | 0.79 | 0.17-2.60 | 0.719 |  |  |  |
| **Respiratory syncytial virus** |  |  |  |  |  |  |
| **Human rhinovirus/enterovirus** | 0.71 | 0.30-1.54 | 0.410 |  |  |  |
| **Influenza A Virus** | 0.41 | 0.12-1.09 | 0.105 | 0.46 | 0.13-1.21 | 0.156 |
| **SARS-CoV-2** | 0.29 | 0.02-1.50 | 0.240 |  |  |  |
| **Parainfluenza Virus** | 0.96 | 0.15-3.62 | 0.953 |  |  |  |
| **SARS-CoV-2** |  |  |  |  |  |  |
| **Human rhinovirus/enterovirus** | **0.20** | **0.03-0.71** | **0.033** | **0.20** | **0.03-0.71** | **0.033** |
| **Influenza A Virus** | 0.13 | 0.01-0.66 | 0.052 | 0.13 | 0.01-0.66 | 0.052 |
| **Respiratory syncytial virus** | 0.28 | 0.02-1.44 | 0.226 | 0.28 | 0.02-1.44 | 0.226 |
| **Parainfluenza Virus** | 0.00 | 0-9e^24^ | 0.992 | 0.00 | 0-9e^24^ | 0.992 |
| **Parainfluenza Virus** |  |  |  |  |  |  |
| **Human rhinovirus/enterovirus** | 1.01 | 0.31-2.89 | 0.985 |  |  |  |
| **Influenza A Virus** | 0.80 | 0.18-2.65 | 0.743 |  |  |  |
| **Respiratory syncytial virus** | 0.96 | 0.15-3.66 | 0.961 |  |  |  |
| **SARS-CoV-2** | 0.00 | 0-2.3e^32^ | 0.991 | 0.00 | 0-2.4e^32^ | 0.991 |

^i^In Model 1, selected respiratory tract pathogens (Prevalence>4%) were entered in the model as explanatory variables. ^ii^In Model 2, a backward logistic regression model was created. Bold and red values indicate statistically significant results.
